# Impact of Different Parameters to Enhanced Corrosion Resistance of Zinc-PVDF-G Coating for the Protection of Mild Steel Substrates

**DOI:** 10.3390/polym17212914

**Published:** 2025-10-31

**Authors:** Saba Ayub, Bashar S. Mohammed, Ahmad Mahamad Al-Yacouby, Naraindas Bheel

**Affiliations:** Department of Civil and Environmental Engineering, Universiti Teknologi PETRONAS, Seri Iskandar 32610, Perak, Malaysia; bashar.mohammed@utp.edu.my (B.S.M.); ahmad.alyacouby@utp.edu.my (A.M.A.-Y.); naraindas_20001014@utp.edu.my (N.B.)

**Keywords:** zinc-PVDF-graphene, nanocomposite coatings, mild steel protection, parameters, corrosion resistance

## Abstract

This study investigates the corrosion resistance of zinc-PVDF-graphene (zinc-PVDF-G) coatings on mild steel substrates. Coatings with varying graphene concentrations were prepared using electrochemical deposition for zinc, followed by brush coating of PVDF-graphene. Central composite design (CCD) of response surface methodology (RSM) was employed to optimise the coating composition, and high R^2^ values confirmed the reliability of the models. Characterisation using scanning electron microscopy (SEM), X-ray diffraction (XRD), and Fourier transmission spectroscopy (FTIR) demonstrated uniform coating morphology and strong adhesion. The inclusion of 0.9% graphene in PVDF significantly enhanced corrosion resistance, with the P3-coated sample achieving a barrier performance of 5.24 × 10^6^ Ω·cm^2^, an OCP of −0.563 V, and a protection efficiency that improved by approximately 61.6% compared to graphene-free coatings. These results indicate that graphene effectively reinforces the PVDF matrix, reduces diffusion of corrosive species, and provides superior long-term stability, demonstrating the potential of zinc–PVDF-G coatings for high-performance corrosion protection.

## 1. Introduction

Corrosion is the most significant and challenging problem in the industry [1]. In industrial manufacturing, the majority of metals and their alloys, including carbon steel, copper (Cu), nickel (Ni), and magnesium, often experience corrosion [2]. Mild steel and carbon steel are extensively employed industrial materials in the fields of chemical processing, petroleum and water pipeline utilities, and marine structures [3]. This is mainly because of its affordability, excellent forming and welding qualities, and wide range of applications [4]. However, the humid environments are particularly harsh for the steel material [5]. Mild steel is a non-alloy steel that is used in pipelines and the construction sectors [6]. Mild steel is composed primarily of iron with very low carbon content (0.05–0.25%) and trace amounts of alloying elements, making it ductile and easy to work with but also highly susceptible to corrosion due to the absence of protective elements such as chromium and nickel [7]. The manufacturing sector has shown increasing interest in zinc and zinc alloy coatings as an effective method for protecting steel products from corrosive environments. Zinc is more readily applied and less expensive to utilize compared to alternative metallic coatings, including tin, chromium, nickel, or aluminium [8]. The two main purposes for using zinc “galvanizing” are as follows: Firstly, it exhibits favourable chemical corrosion resistance; secondly, when exposed to an electrolyte, zinc corrodes preferentially to steel [9].

Several coating methods are used, including electrochemical deposition, heated dipping, etc. Electrochemical deposition of zinc produces a thinner coating than heated dipping, rendering it more suited for subsequent forming operations within the automotive industry [10]. These coatings extend the service life of the metal by offering protection to the steel against corrosion [11]. The use of nanoparticles, particularly graphene and other carbon materials, has drawn a lot of interest lately for improving the mechanical and corrosion-resistant qualities of coatings [12,13]. In addition to their aesthetic qualities, these coatings will offer advantageous mechanical characteristics, including microhardness, abrasion resistance, ductility, and strength. When electrodeposited from a solution containing Zn^2+^ ions, the Zn layer applied to the substrate is typically denser. Several studies have been conducted to investigate the factors that make the zinc coating process distinctive, including parameters such as pH, bath composition, voltage, and current. However, recent research suggests that combining zinc with a graphene layer can enhance the corrosion protection properties even further [14].

Graphene is the thinnest 2D carbon substance known to science. Both the scientific and industrial communities have taken an interest in single-layer graphene ever since Geim and Novoselov first used a micro-mechanical stripping approach to create it [15]. Graphene is among the most effective materials against corrosion because it possesses hydrophobic characteristics and excellent impermeability. Due to its dense atomic structure, pure graphene serves as an exceptional barrier against oxygen, water, and other corrosion-causing substances. When corrosion starts at the metal–coating interface, electrons move from anodic sites to cathodic sites through the metal, driving the corrosion reaction [16,17,18,19]. But before graphene coatings are used to protect enormous structures from corrosion or to produce vast quantities of items like steel rolls, etc., several challenges must be resolved [20]. First, the loss of impermeability and concentration cell development at the graphene sheet defect location led to accelerated corrosion of the underlying material. Another significant technical issue is the transfer of graphene sheets to foreign surfaces and their long-term adherence to such surfaces under service circumstances [21]. To overcome these challenges, graphene and graphene oxide (GO) can also be utilised to prevent corrosion by being incorporated into metal particles, which are conventional protective metallic coatings [22,23]. Another promising approach is using graphene as a filler within a polyvinylidene fluoride (PVDF) matrix. PVDF exhibits excellent thermal and mechanical properties, exceptional hydrophobicity, and outstanding resistance to fatigue, stress fracture, erosion, and corrosion, making it effective for protecting metals and alloys in harsh environments [24,25]. Nevertheless, the presence of unbound volumes within the PVDF matrix leads to the development of diverse cracks or abrasions that compromise the integrity of the protective film. This compromise enables oxygen, water, and ions to pass through, thereby facilitating the metallic surface’s exposure to the corrosive medium [26].

To address this limitation, polymeric coatings have been supplemented with nanofillers, including metals, carbonaceous compounds, or metal oxide nanoparticles (NPs), to produce nanocomposites that effectively protect metallic surfaces against corrosion [27,28]. The corrosion resistance properties can be improved using nanofillers, which decrease contact tension or wettability as well as water and aggressive ion penetration by elongating the sinuous pathway and reducing surface roughness. Xie et al. investigated the potential of GO as an accelerator in the zinc phosphate process on steel. Both the microstructure and corrosion resistance of phosphate coatings were investigated in relation to GO. The results of the study indicated that GO sheets operated as sedimentary deposits, which facilitated the accumulation of phosphate crystals and the capture of metal ions. At an optimum GO concentration of 1.2 g/L, the most uniform structure and greatest resistance to corrosion were observed. This demonstrates the potential for 2D nanomaterials such as GO to improve phosphating processes and prevent corrosion [29]. Another study also reported that the composite EP/iDCNTs/Zn/PVDF with the inclusion of nanoparticles within the polymer matrix enhanced the anti-corrosion properties [30]. It has also been reported that the Zn-GO composite coating electrodeposited was used to protect the mild steel substrate [31,32]. Zinc has a positive impact because zinc acts as an anodic sacrificial; when in contact with steel, it has anti-corrosive properties [31].

Therefore, this work investigates the use of a graphene-modified PVDF (PVDF-G) composite as a secondary corrosion protection layer over a primary zinc coating. Although few studies have used polymer metal oxides to investigate the corrosion, achieving long-term durability and an impermeable barrier remains challenging. Graphene, with its atomic-level impermeability, hydrophobic nature, and excellent mechanical strength, offers unique advantages over conventional metal oxide fillers [32].

Therefore, in this study, we explore the use of a polyvinylidene fluoride–modified graphene (PVDF-G) composite as an innovative secondary corrosion protection layer applied over a primary zinc coating. This work is in continuation of the previous work [33], where we utilised different parameters to deposit zinc coating on mild steel for corrosion resistance. This dual-layer strategy is designed to significantly enhance corrosion resistance by combining the sacrificial protection provided by zinc with the superior barrier properties and mechanical strength of the PVDF-G composite. Here, PVDF acts as a durable barrier that synergistically complements zinc and graphene, improving the overall anti-corrosion performance. The interaction between these layers is expected to provide a more effective and long-lasting defence against corrosive environments compared to conventional coatings.

## 2. Materials and Methods

### 2.1. Materials

Polyvinylidene fluoride (PVDF) pellets (Mw = 180,000 g/mol, density 1.78 g/cm^3^, Sigma-Aldrich, Petaling Jaya, Malaysia) were dissolved in N-methyl-2-pyrrolidone (NMP) to prepare the polymer solution. Graphene (molecular weight 12.01 g/mol) was used. All materials were purchased from Sigma-Aldrich and used as received without further purification.

### 2.2. Samples Preparation of Zn-PVDF-G Coating and Methods

The testing sample used in this study is a mild steel sheet (2 × 2 cm^2^). The materials are zinc sheets (3 × 2 cm^2^), graphene grade H with a thickness of 15 nm, sodium sulphate (Na_2_SO_4_), zinc sulphate (ZnSO_4_), sodium chloride (NaCl), and deionised water. The prepared electrolyte composition is detailed in Table 1.

For coating, the sample of mild steel was polished using a range of finer grades of emery paper (P400) before electroplating. After dipping the sample of the mild steel into the 0.5 M HCl solution and letting it sit for ten minutes, it was rinsed with distilled water. The cathode and anode were both connected to a direct current (DC) power supply. Brightener, zinc sulphate, sodium sulphate, and sodium chloride were utilised as chemical reagents. To determine the optimal thickness of the zinc coating applied as the first layer, 9 samples were prepared and tested, varying two factors: voltage (ranging from 3 V to 10 V) and coating time (15, 30, and 45 min). The results indicated that the sample coated at 10 V for 45 min achieved the highest average thickness of 640 microns, as detailed in Table 2. Therefore, 10 V is used for 45 min to coat zinc in an electrolyte, and three samples were prepared as p1, p2, and p3. As a next step, the PVDF pellets (4 g) were dissolved in approximately 50 mL of N-methyl-2-pyrrolidone (NMP) by heating at 90 °C and stirring at 500 rpm for 45 min to obtain a homogeneous solution. Graphene was then introduced into the solution in varying amounts (0.3 g, 0.6 g, and 0.9 g), corresponding to 0.6 wt%, 1.2 wt%, and 1.8 wt%, resulting in three distinct mixtures. Ultrasonication was employed for 120 min to ensure thorough mixing. Each mixture (containing 0.3 g, 0.6 g, and 0.9 g of graphene) was subsequently applied to the pre-prepared samples, labelled p1, p2, and p3, using a simple brush. The coated samples were left to dry at room temperature for 12 h.

Several tests were employed for characterisation, such as X-ray diffraction (XRD) analysis, which was carried out in the diffraction range of 10–80° to determine the crystallinity and phase composition of the manufactured material samples. A scanning electron microscope (SEM) was used to examine the morphological composition of the coated samples. The sample’s functional groups were investigated using Fourier transform infrared spectroscopy (FTIR). The coating thickness values reported in Table 2 were measured using an Elcometer 456 Digital Coating Thickness Gauge. For each sample, measurements were taken at five different locations, and the average value is presented in the table. Electrochemical impedance spectroscopy (EIS) was used to analyse the coated samples’ corrosion behaviour. Prior to the electrochemical experiments, the samples were immersed in a 3 wt.% NaCl corrosive solution to achieve an ideal open circuit potential (OCP) at room temperature, and then the OCP and polarisation curves were measured at different immersion times, such as 1 h, 24 h, and 48 h.

In the last stage, the response surface methodology (RSM) method was employed to investigate the impacts of various factors and their combinations on the coating preparation of the samples. To investigate the corrosion characteristics of zinc-PVDF-G at three graphene concentrations, using the RSM central composite design (CCD) method, the different experimental designs were created, with different proportions and five randomly repeated occurrences of each component. To check the authenticity of the research and account for any potential changes, the duplicate samples were used. Corrosion resistance and other physical properties were among the element input relationships that the RSM also evaluated. The input factors are selected for designing the RSM modelling to optimise the output responses (EIS, OCP, and BMP) as shown in Table 3.

## 3. Results and Discussion

### 3.1. EIS Measurement of the Zinc-PVDF-G Coated Samples

The anti-corrosion performance of the examined coatings was confirmed using EIS immersed in 3% NaCl Solution. While other corrosion tests were not performed, the EIS and OCP results provide robust evidence of the enhanced corrosion protection of the zinc–PVDF-graphene coating.

**Figure 1:**
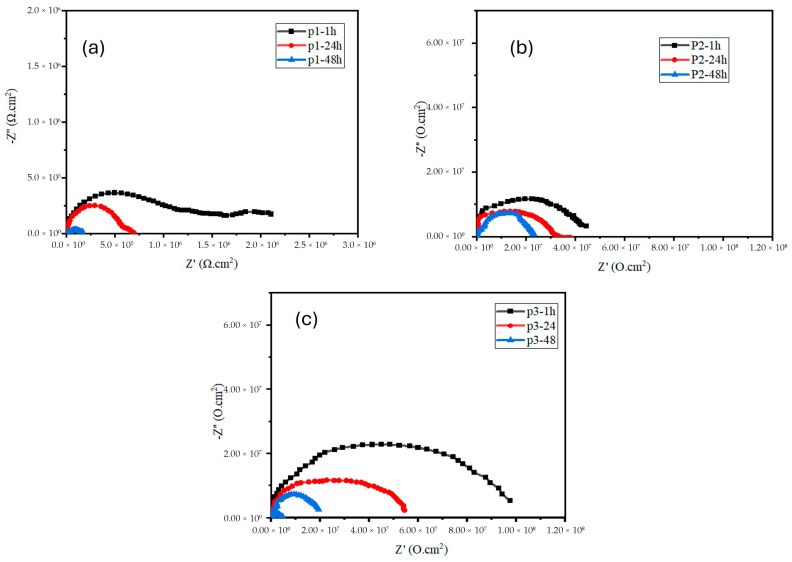
Nyquist plot for zinc-PVDF-G samples (**a**) P1, (**b**) P2, and (**c**) P3 in different immersion times.

Figure 1 shows EIS spectra representing Nyquist plots of the zinc-PVDF-G Samples. Figure 1a represents the sample p1 at different immersion times. It can be observed that, after 1 h of immersion time, it shows a better semi-circle. Figure 1b represents the p2 sample. It can be observed that, as the concentration of graphene increases in this sample, the greater semicircle diameter can be observed after 1 h of immersion time. Similarly, in Figure 1c, it can be observed that the semicircle diameter is wider at different immersion times as compared to p1 and p2. In general, a coating with a bigger semicircle diameter or a better charge transfer resistance indicates a lower rate of corrosion [34]. Overall, the reduced inhibition after 1 hr and the distorted semicircles are behaviours that correspond to the initial wetting and equilibration of the coating in the corrosive medium. After this short period, the coating stabilises and the impedance increases, indicating the establishment of an effective protective barrier. The reduction in corrosion rate that is seen when charge transfer resistance increases is thought to be caused by either the creation of corrosion products or the mitigation of corrosion processes at the active sites located under the coating [35]. The improved corrosion resistance in samples with higher graphene content (p2 and p3) is attributed to graphene’s excellent barrier properties, which prevent the penetration of corrosive agents like chloride ions.

Graphene also helps distribute electrochemical potential, reducing localised corrosion and promoting the formation of protective films that fill micro-defects. Over time, while all samples show a decline in impedance due to electrolyte infiltration, the p3 sample, with the highest graphene concentration, retains superior protection. This highlights graphene’s role in extending the lifespan of coatings by enhancing durability and charge transfer resistance, even after prolonged exposure to corrosive environments.

To assess the role of PVDF and graphene with zinc, the corrosion performance of only zinc coating, as reported in the literature [36], was considered. Zinc coatings typically exhibit a barrier performance of around 350 Ω·cm^2^, while zinc + PVDF coatings improve protection by ~Z%. In comparison, the zinc-PVDF-graphene coatings in this study demonstrate significantly higher impedance, indicating that graphene incorporation effectively reinforces the polymer matrix and enhances long-term corrosion protection.

**Figure 2:**
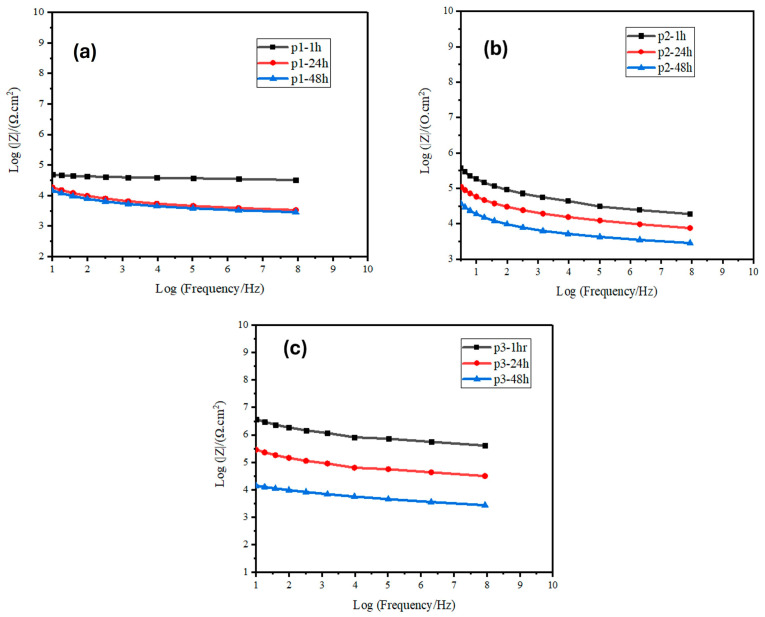
Bode module plots of zinc-PVDF-G samples (**a**) p1, (**b**) p2 and (**c**) p3 in different immersion times.

To evaluate the anticorrosion behaviour, the critical impedance modulus at the lowest frequency of the Bode diagram (|Z|0.01 Hz) is a crucial parameter, which is mentioned in the literature [37]. Figure 2a illustrates the p1 sample in the Bode module plot of the coatings. The sample exhibits a gradual decrease in the value of |Z|, suggesting that the corrosive medium might penetrate the interface of the coating and initiate a reaction with the substrate. Conversely, Figure 2b,c illustrate the p2 and p3 coating samples, where the initial |Z|0.01 Hz value of the coating increased by an order of magnitude when more graphene concentration was added. Furthermore, an additional characteristic that was observed in all the coating samples is presented in Figure 2. After 48 h of immersion, the value of |Z|0.01 Hz decreased progressively. The enhanced corrosion resistance is attributed to the physical barrier effect provided by the graphene. Initial low-frequency impedance moduli were greater for composite coatings containing zinc-PVDF-G functional filler. The modulus of low-frequency impedance for composite coatings decreased gradually after 48 h of immersion time. Despite a gradual decrease in low-frequency impedance values after 48 h of immersion across all samples, the coatings containing graphene (p2 and p3) maintain higher |Z|0.01 Hz values compared to p1. This indicates that the zinc-PVDF-G composite coatings provide more durable protection, even after prolonged exposure to the corrosive environment. Graphene’s inclusion thus plays a pivotal role in delaying the onset of corrosion, enhancing the longevity and efficacy of the coatings.

**Figure 3:**
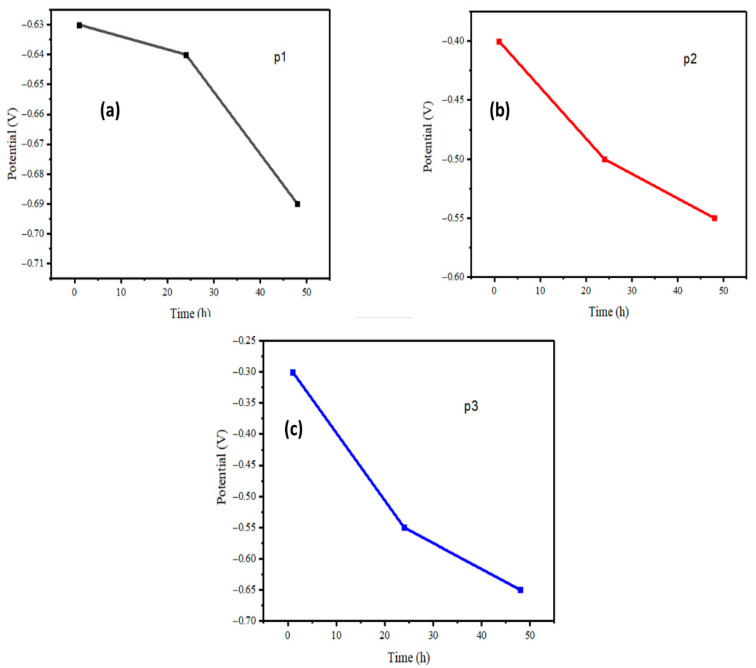
Average OCP evaluation plots of zinc-PVDF-G samples (**a**) p1, (**b**) p2 and (**c**) p3 in different immersion times.

As seen in Figure 3, the average OCP value points on the graph indicate that the prepared coating samples exhibited a high OCP value during the initial immersion period for each sample. The p1 sample is illustrated in Figure 3a, where the initial OCP value is −0.63 after 1 h of immersion time and gradually decreases after 24 h of immersion time. The coating sample p2 OCP is depicted in Figure 3b. The value of OCP is observed to be −0.4 after 1 h of immersion, and after 24 h, −0.50 further decreases after 48 h of immersion time. The OCP of coating sample p3 is depicted in Figure 3c. Following a 1 h immersion period, the sample exhibited the highest OCP value of −0.30, surpassing all other samples. After 48 h of immersion time, the value decreased. The higher initial OCP values for p2 and especially p3 suggest that the increased graphene content significantly enhances the coatings’ initial corrosion resistance. However, the overall decline in OCP for all samples over time indicates the gradual penetration of the corrosive medium into the coatings, reducing their protective performance. Despite this, p3 remains the most effective in retaining corrosion resistance over extended immersion periods. Although the OCP of the P3 sample shows a more pronounced decrease during immersion, its final potential after 48 h is comparable to that of P2. Since the open-circuit potential alone cannot serve as a definitive indicator of corrosion resistance, the overall assessment is based on the impedance behaviour. The EIS results reveal that P3 maintains a larger semicircle diameter and higher impedance values throughout the immersion period, indicating superior barrier characteristics and sustained protection against corrosion.

### 3.2. XRD Analysis

**Figure 4:**
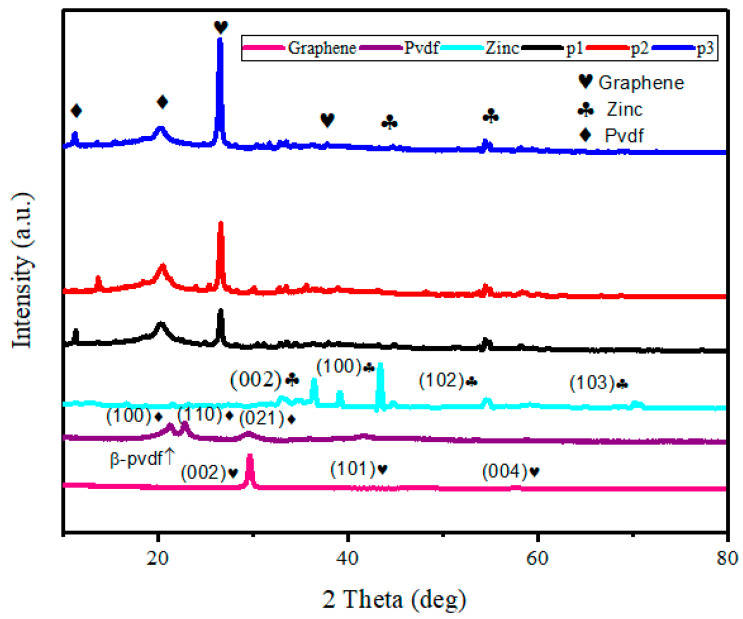
XRD graph of graphene, PVDF, zinc, and zinc-PVDF-G samples.

The XRD graphs of graphene, PVDF, zinc, and the p1, p2, p3 samples are shown in Figure 4. The XRD pattern of the graphene product exhibits one prominent peak, approximately 2θ = 28° and 2θ = 43.2°. These carbon peaks have been attributed to the (002) and (101) planes [38,39], whereas 2θ = 56.70° belongs to the plane (004) [40]. The PVDF graph shows the prominent peaks around 2θ = 21° belong to the plane (100), which indicates the beta phase [41]. The other two peaks at 2θ = 22.94° and 2θ = 29.35° belong to the plane (110) and (021), respectively [42]. The zinc graph shows the peaks at 2θ = 36.52°, 39.36°, 54.55°, 70.31° belong to the plane (002), (100), (102), (103), respectively [43]. Overall, it can be observed in samples p1, p2, and p3 that, as the concentration of graphene increases, the carbon peak intensity increases and the beta phase also becomes more prominent, and these samples clearly indicate the presence of graphene, zinc, and PVDF in the coated samples. In the composite samples (p1, p2, p3), an increase in graphene concentration is evidenced by the corresponding increase in the intensity of the carbon peaks. Additionally, the β-phase of PVDF becomes more pronounced with higher graphene content, as seen by the stronger peak at 2θ = 21°. This suggests that graphene incorporation enhances the crystallinity and phase composition of PVDF in the coating. Overall, the XRD patterns confirm the successful incorporation of graphene, zinc, and PVDF in the p1, p2, and p3 coatings, with increasing graphene content positively influencing the structure and properties of the samples.

### 3.3. SEM Analysis

**Figure 5:**
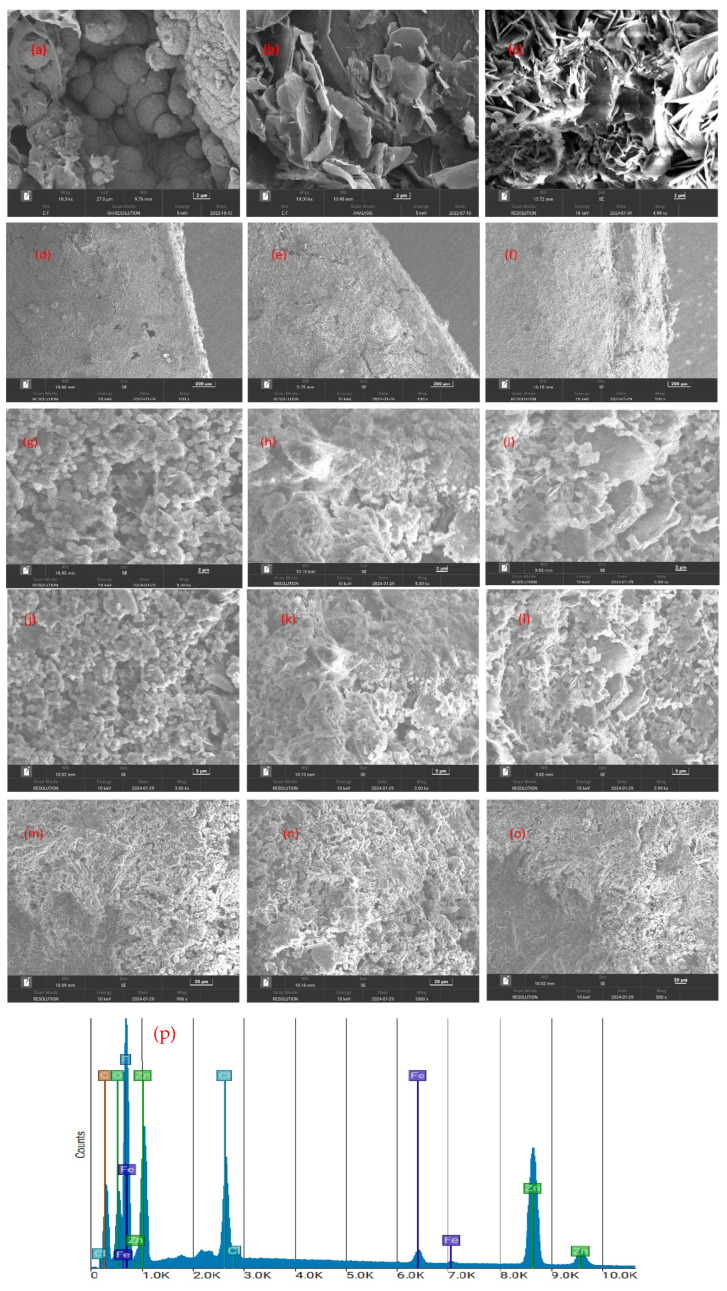
SEM images of zinc-PVDF-G Samples: (**a**) PVDF, (**b**) graphene, (**c**) zinc, (**d**) p1 sample coated edge, (**e**) p2 sample coated edge, (**f**) p3 sample coated edge, (**g**) p1 at 2 µm, (**h**) p2 at 2 µm, (**i**) p3 at 2 µm, (**j**) p1 at 5 µm, (**k**) p2 at 5 µm, (**l**) p3 at 5 µm, (**m**) p1 at 20 µm, (**n**) p2 at 20 µm, (**o**) p3 at 20 µm, (**p**) EDS image of p3 sample.

Figure 5 represents the structural morphology of graphene, PVDF, zinc, and the coated samples, p1, p2, and p3. As can be observed in Figure 5a, the surface morphology of the PVDF particles was bonded to each other [44]. Figure 5b shows the graphene structure as it appears in three-dimensional crumpled morphology. The zinc surface image depicted in Figure 5c illustrates its lotus leaf-like morphology. The surface edges of the coated samples p1, p2, and p3 are depicted in Figure 5d, e, and f, respectively. The edge surfaces of p1 and p2 are finer in appearance compared to the p3-coated sample. Figure 5g–i illustrate the surface morphology at 2 µm. Figure 5g illustrates the close inter-particle encapsulation of PVDF and the relatively uniform distribution of nanoparticles. As shown in Figure 5h, which depicts the p2-coated samples, graphene and zinc particles aggregated in this sample. This may be the result of an increase in graphene concentration. The samples p3 illustrate the distribution of graphene within the PVDF matrix, as depicted in Figure 5h. The findings suggest that, as the graphene content increases, the agglomeration of the nanoparticles becomes more prominent, which is accompanied by a more unequal dispersion, but the coating still exhibits superior corrosion resistance. This is attributed to the enhancement of PVDF crystallinity, the creation of a tortuous path for corrosive species, and strong graphene-PVDF interfacial interactions, which collectively maintain barrier performance [45].

Figure 5j–l show the structural morphology at 5 µm, and Figure 5m–o represent the morphological structure at 20 µm, whereas Figure 5p shows the EDS graph of the p3-coated sample. In conclusion, as the graphene content increases in the samples, there is a greater tendency for particle agglomeration, leading to a less uniform distribution, which may affect the mechanical and corrosion-resistant properties of the coatings. However, the presence of graphene enhances the overall structural integrity, as indicated by the morphology and elemental distribution in the samples.

### 3.4. FTIR Analysis

**Figure 6:**
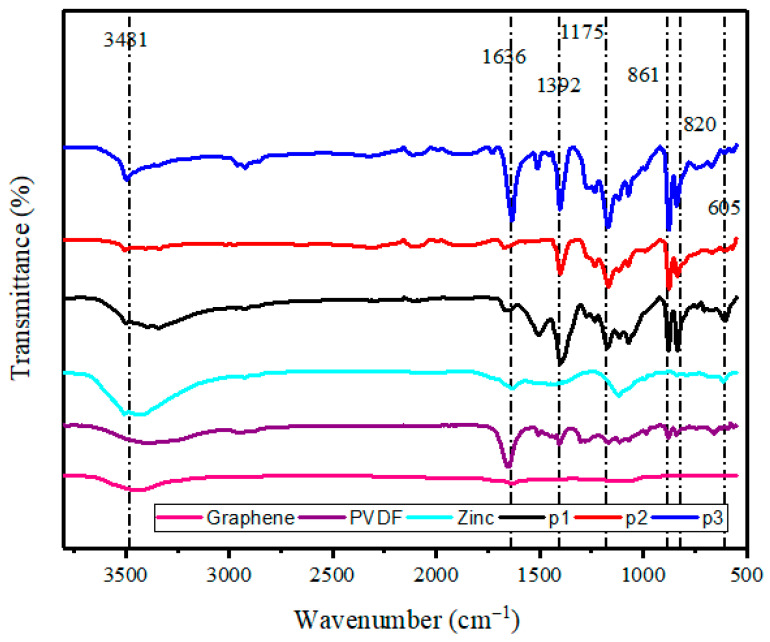
FTIR graph of graphene, PVDF, zinc, and zinc-PVDF-G samples.

Figure 6 shows the FTIR spectra of the graphene, PVDF, zinc, p1, p2, and p3 samples. It can be seen in Figure 6 that the PVDF peaks show at 1392 cm^−1^ and 1175 cm^−1^, which belong to the C-H and C-F stretching and deformation [45]. These peaks are prominent in PVDF-, p1-, p2-, and p3-coated samples. The peaks at 3481 cm^−1^ and 1636 cm^−1^ correspond to the O-H and C=C Stretching vibrations [46]. The presence of two peaks at 820 cm^−1^ and 860 cm^−1^ corresponds to the α and β phases, respectively. It is evident that the incorporation of graphene filler results in a reduction in the intensity of the alpha phase, while the intensity of the beta phase increases [47]. The presence of an absorption band at 605 cm^−1^ can be attributed to the stretching mode of zinc. The intensity and position of these peaks change minimally among the coated samples, even when PVDF with graphene is applied onto a substrate composed of zinc. This indicates that the coating process did not significantly alter the structure and crystalline phase of the zinc particles [48]. Overall, the incorporation of graphene into the zinc-PVDF matrix enhances the β-phase of PVDF while maintaining the structural integrity of the zinc particles, as evidenced by the minimal changes in peak positions and intensities across the samples. This indicates that the coating process successfully retains the desired structural characteristics of both PVDF and zinc, while benefiting from the enhanced properties provided by the addition of graphene.

### 3.5. Response Surface Methodology Modelling and Optimisation

Response surface modelling (RSM) is a statistically driven optimisation strategy that replaces expensive “black-box” processes with an inexpensive polynomial proxy. It does so by selecting a compact, purpose-built experimental design, most often a central composite or Box–Behnken layout. Data are then collected, a quadratic model linking controllable factors to the measured response is fitted and diagnosed, and the model is interrogated to locate the stationary point or ridge that maximises or minimises the desired output. Because the fitted surface can be visualized as contours or 3D plots and updated sequentially, researchers can navigate toward optimum operating conditions with a minimal number of runs, verify predictions through confirmation experiments, and rapidly re-optimise whenever specifications or constraints change.

Empirical models that predict the response were built and validated against the experimental results. Depending on how the inputs interact and influence the response, the model adopts either the linear form of Equation (1) or the quadratic form of Equation (2).

(1) $$y=\beta_{0}+\beta_{1}x_{2}+\beta_{2}x_{2}+\beta_{n}x_{n}+\epsilon \;$$

(2) $$y=\beta_{0}+\sum_{i=1}^{k}\beta_{i}x_{i}+\sum_{i=1}^{k}\beta_{ii}x_{i}^{2}+\sum_{j=2}^{k}\sum_{i=1}^{j=1}\beta_{ij}x_{i}x_{j}+\epsilon \;$$

The symbol y denotes the predicted response; xi and xj are the coded input variables, the subscripts i and j index their linear and quadratic terms, β0 is the model’s y-intercept, k is the total count of independent factors, and ε is the residual error.

#### 3.5.1. Analysis of Variance (ANOVA)

The Equations (3)–(5) provide the models for all output parameters. Based on the preferred sequential model sum of squares (SMSS), quadratic models were better for BMP and OCP evaluation because they included more meaningful extra terms and were not aliased. However, the EIS linear model was based on SMMS. Presented models use coded factors, representing input factors’ lowest, intermediate, and greatest values as −1, 0, and +1.

(3) $$BMP=+6.70-1.45\times A+0.41\times B+0.84\times AB+1.61\times A^{2}+0.54\times B^{2}$$

(4) $$OCP=-0.50-0.070\times A+0.057\times B-0.044\times AB+0.013\times A^{2}-0.11\times B^{2}$$

*EIS* = +6.575 × 10^6^ − (3.654 × 10^5^) × *A* + (4.199 × 10^6^) × *B* (5)

The hours are indicated by A, and the filler (graphene) is indicated by B. Moreover, EIS stands for electrochemical impedance spectroscopy, OCP stands for open circuit potential, and BMP stands for Bode module plots.

An ANOVA test was conducted on the models with a 95% confidence range. This meant that models and terms with a chance of less than 5% were thought to be significant. The ANOVA findings have been detailed in Table 4. With a likelihood value (*p*-value > F) of less than 0.005, all the models that were made are noteworthy. The model terms are marked as either significant or insignificant based on whether their *p*-value > F is less than or greater than 5%, as shown in Table 4. On the other hand, for the model to fit, the *p*-value for the “Lack of fit” needs to be “insignificant” (>5%). So, out of all the types that were made, only the BMP does not fit very well. But when the troubleshooting tools for other models are considered, even the BMP model is found to be strong enough for this task.

Table 5 shows some other model validation factors that were taken into account in the study. It is clear that all of the models have a fairly high coefficient of determination (R^2^). This number shows how well the model fits the real-world data, where the R^2^’s number ranges from 0 to 1, or 0% to 100% [49]. A value of 1 or very close to 1 is ideal. All of the models had an R^2^ value between 66 and 98%, which means that the picked models fit the data well. Also, the modified R^2^ and projected R^2^ should not be more than 0.2 apart for the model to be very good at predicting the reaction. From the Adj. R^2^ and Pred. R^2^ numbers for all the models, we can see that this condition is met; however, there are a few variations in R^2^ and Pred. R^2^ arises from minor deviations in the coating behaviour during initial immersion and small experimental noise. Furthermore, a developed model needs to have an adequate precision (Adeq. Precision) of more than four. All of the developed models have an adequate precision of more than ten, which shows that they are strong and can correctly predict the answers.

#### 3.5.2. Model Diagnostics and Response Surface Plots

**Figure 7:**
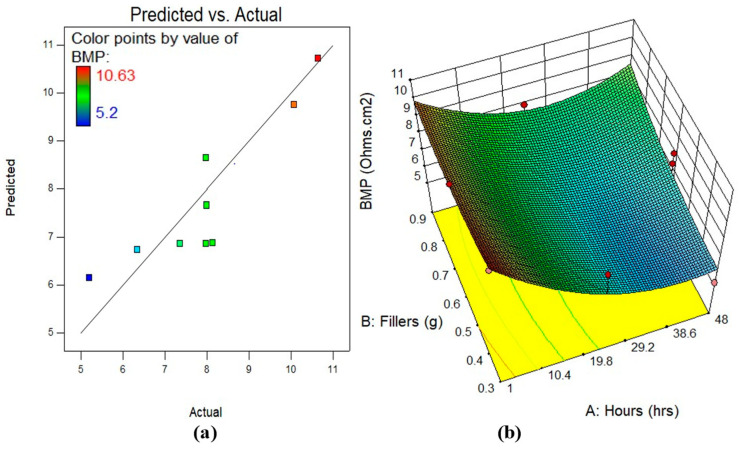
(**a**) Predicted against actual plots, (**b**) the 3D surface diagram for BMP analysis.

**Figure 8:**
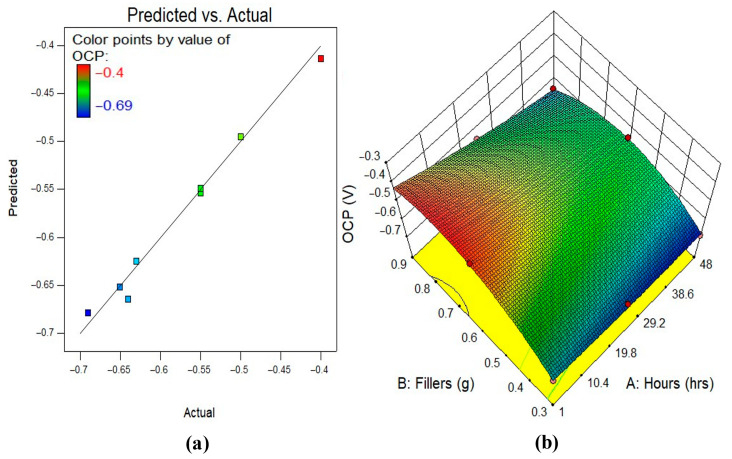
(**a**) Predicted against actual plots, (**b**) the 3D surface diagram for OCP analysis.

**Figure 9:**
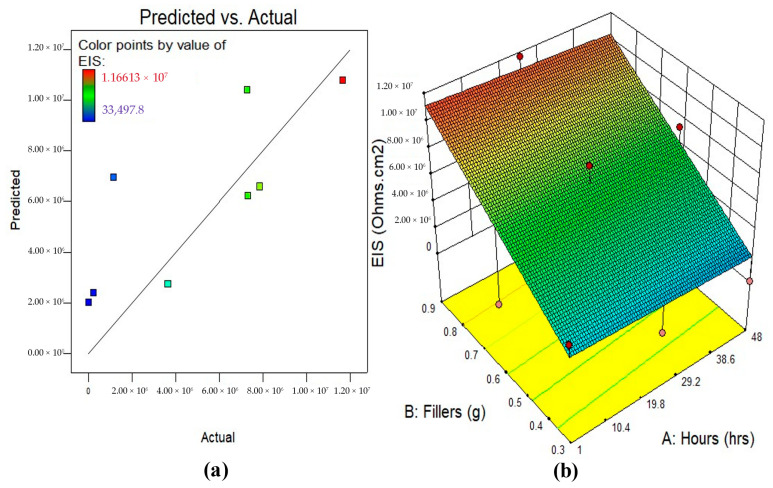
(**a**) Predicted against actual plots, (**b**) the 3D surface diagram for EIS analysis.

A 2D contour plot or 3D response surface plot may better show the response’s variable interaction. The 3D response surface plot shows the same data as the 2D contour plot. Figure 7, Figure 8 and Figure 9 illustrate the actual vs. anticipated diagnostic plot and 3D response surface diagrams of all response-predicting models in this study. As seen in the actual vs. projected plots, all constructed models have data points completely aligned to the line of fit, indicating a strong correlation between experimental data and model response data. Colour-coding represents regions on 3D response surface diagrams that reflect response intensities from input variable interaction. The greatest reaction intensities are in red, while the lowest are in blue. The figures show that the input factors’ interactions and effects on responses match the preceding talks on the variables’ effects on BMP, OCP, and EIS evaluation.

#### 3.5.3. Multi-Objective Optimisation

Finding the ideal value for all the variables so that they provide the best possible result is the goal of optimisation. Goals are established for the input and output variables (factors) with different criteria and levels of significance in order to accomplish the intended purpose. The optimisation is assessed using the attractiveness value, which ranges from 0 to 1. A closer approximation to one indicates a better result.

**Figure 10:**
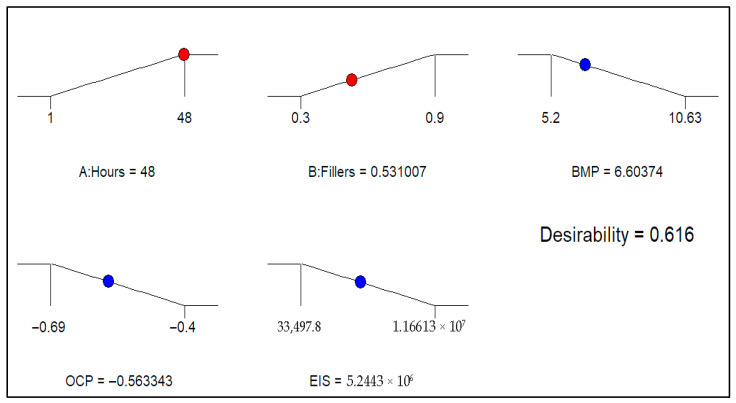
Optimisation solution ramps.

**Figure 11:**
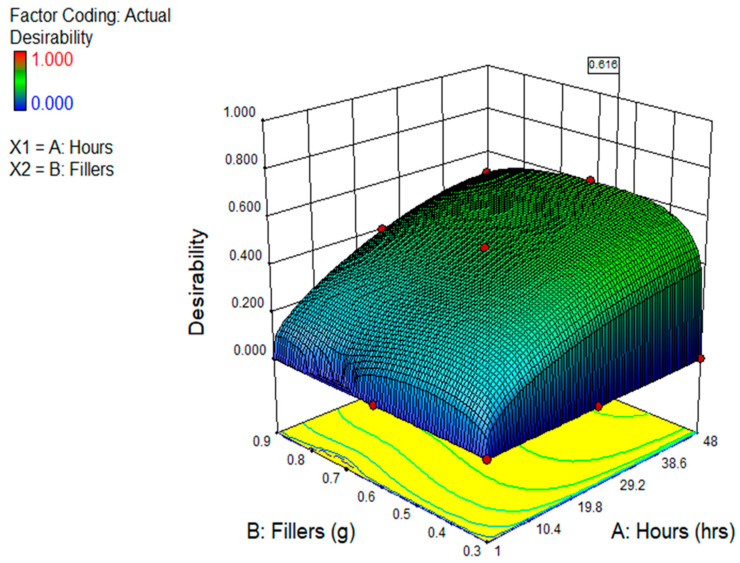
The 3D diagram of desirability factor of the study.

Table 6 illustrates the findings and also indicates that the optimisation’s objective functions were constructed in this case. By adjusting the input variables to 48 h and adding 0.53 g of graphene, the result shows that an ideal BMP, OCP, and EIS of 6.60 Ohms.cm^2^, −0.563 V, and 5.2443 × 10^6^ Ohms.cm^2^ could be achieved, with a high desirability value of 61.60%. Figure 10 displays the optimisation solution ramps, while Figure 11 displays the desirability findings.

## 4. Conclusions

In summary, zinc-PVDF-G coatings were successfully prepared using electrochemical deposition and brush coating techniques. Among the samples, P3 exhibited the highest corrosion resistance, with the inclusion of 0.9% graphene significantly enhancing the barrier properties of the zinc-PVDF-G. This improvement is attributed to graphene’s atomic-level impermeability and reinforcement of the polymer matrix, which increases coating thickness and effectively hinders the diffusion of water, oxygen, and corrosive ions.

The RSM model and ANOVA analysis confirmed that the selected concentrations of graphene and PVDF were optimal for achieving maximum corrosion protection. These results demonstrate that controlled composition and coating design can substantially improve the long-term stability and performance of mild steel substrates, providing a quantitative and mechanistic understanding of how graphene incorporation enhances corrosion resistance. For every model, the gap between adjusted and predicted R^2^ stays below 0.2, and both statistics meet or exceed the 95% confidence threshold.

## Figures and Tables

**Table 1:** The prepared electrolyte composition.

Material	Composition
Na_2_SO_4_	3.75 g
NaCl	1.75 g
ZnSO_4_	22.5 g
Deionized water	250 ml

**Table 2:** Coating thickness of the prepared samples at different parameters.

Sample	Voltage (V)	Time (min)	Average Measured Thickness (µm)
CZ1	3	15	64.9
CZ2	3	30	109
CZ3	3	45	260
CZ4	5	15	411
CZ5	5	30	489
CZ6	5	45	501
CZ7	10	15	612
CZ8	10	30	627
CZ9	10	45	640

**Table 3:** Input factors selected for RSM modelling.

S.No	Input Factors	Ranges
1.	Hours	1 to 48
2.	Graphene	0.30 to 0.90

**Table 4:** Analysis of variance (ANOVA) result.

Response	Source	Sum of Squares	Df	Mean Square	F-Value	*p*-Value > F	Significance
BMP	Model	41.22	5	8.24	15.04	0.0002	significant
	A-Hours	9.03	1	9.03	16.47	0.0023	significant
	B-Fillers	0.84	1	0.84	1.53	0.2449	Not significant
	AB	1.65	1	1.65	3.01	0.1134	not significant
	A^2^	6.55	1	6.55	11.95	0.0062	significant
	B^2^	0.97	1	0.97	1.78	0.2123	Not significant
	Residual	5.48	10	0.55	-	-	-
	Lack of Fit	5.29	2	2.64	110.06	<0.0001	significant
	Pure Error	0.19	8	0.024	-	-	
	Cor Total	46.70	15			-	
OCP	Model	0.089	5	0.018	158.35	<0.0001	significant
	A-Hours	0.021	1	0.021	189.18	<0.0001	significant
	B-Fillers	0.016	1	0.016	145.85	<0.0001	significant
	AB	4.429 × 10^−3^	1	4.429 × 10^−3^	39.42	<0.0001	significant
	A^2^	4.252 × 10^−4^	1	4.252 × 10^−4^	3.78	0.0804	Not significant
	B^2^	0.042	1	0.042	373.37	<0.0001	significant
	Residual	1.124 × 10^−3^	10	1.124 × 10^−4^	-	-	-
	Lack of Fit	1.124 × 10^−3^	2	5.619 × 10^−4^	-	-	-
	Pure Error	0.000	8	0.000	-	-	-
	Cor Total	0.090	15	-	-	-	-
EIS	Model	1.286 × 10^14^	2	6.431 × 10^13^	12.64	0.0009	significant
	A-Hours	9.179 × 10^11^	1	9.179 × 10^11^	0.18	0.6779	Not significant
	B-Fillers	1.174 × 10^14^	1	1.174 × 10^14^	23.08	0.0003	significant
	Residual	6.612 × 10^13^	13	5.086 × 10^12^	-	-	-
	Lack of Fit	6.612 × 10^13^	5	1.322 × 10^13^	-	-	-
	Pure Error	0.000	8	0.000	-	-	-
	Cor Total	1.947 × 10^14^	15		-	-	-

**Table 5:** Validation parameters.

Model Validation Constraints	BMP	OCP	EIS
Std. Dev.	0.74	0.011	2.255 × 10^6^
Mean	7.90	−0.56	6.054 × 10^6^
C.V. %	9.38	1.89	37.25
PRESS	47.52	9.380 × 10^−3^	1.120 × 10^14^
−2 Log Likelihood	28.26	−107.61	510.20
R-Squared	0.8826	0.9875	0.6605
Adj R-Squared	0.8240	0.9813	0.6082
Pred R-Squared	−0.0176	0.8959	0.4247
Adeq Precision	10.099	40.877	8.981

**Table 6:** Multi-objective optimisation criteria and solutions.

Factors		Input Factors		Responses (Output Factors)		
		Hours (h)	GO (g)	BMP (Ohms·cm^2^)	OCP (V)	EIS (Ohms·cm^2^)
Value	Min.	1	0.30	5.20	−0.69	33,497.80
	Max	48	0.90	10.63	−0.40	1.16613 × 10^7^
Goal		Maximum	maximum	Minimum	Minimum	Minimum
Results		48	0.531	6.604	−0.563	5.2443 × 10^6^
Desirability		0.616 (61.60%)				

## Data Availability

The original contributions presented in this study are included in the article. Further inquiries can be directed to the corresponding author.
